# Infection by *Rickettsia felis* in *Ctenocephalides felis felis* Fleas from North of Colombia

**Published:** 2019-03-30

**Authors:** Verónica Contreras, Andrés F. Londoño, Jorge Miranda, Salim Mattar, Leidy Y. Acevedo-Gutiérrez, Francisco J. Diaz, Juan D. Rodas

**Affiliations:** 1 Instituto de Investigaciones Biológicas del Trópico, Universidad de Córdoba, Colombia; 2 Grupo de Investigación en Ciencias Veterinarias, Centauro, Universidad de Antioquia, Colombia; 3 Grupo de Inmunovirología, Universidad de Antioquia, Colombia

**Keywords:** *Rickettsia felis*, Fleas, Dogs, Rodents, *Ctenocephalides felis*

## Abstract

**Background:**

*Rickettsia felis* is an emergent Rickettsial agent whose main vector is *Ctenocephalides felis*, but ticks, mites and lice are also infected. We aimed to search for molecular evidence of *Rickettsia* spp. in fleas collected from dogs and wild rodents (Heteromys anomalous) from three villages of Córdoba and Antioquia provinces (Northern of Colombia), where outbreaks of rickettsioses have occurred, and discuss the possible role of fleas on endemic/enzootic regions for rickettsia.

**Methods:**

During 2010 and 2012, 649 *Ctenocephalides felis felis* and 24 *Pulex irritans* fleas were removed from dogs and wild rodents (*Heteromys anomalous*), respectively, in 3 locations from Córdoba and Antioquia provinces (Colombia). These fleas were tested into pools for Rickettsial infection by PCR, targeting *gltA*, *ompB,* and *ompA* Rickettsial genes.

**Results:**

Almost 20% (30/153) of *C. felis felis* pools contained Rickettsial DNA. The fragments of *ompB* gene showed high identity values between sequences from Necocli and Los Cordobas with *R. felis* strain from Senegal (100% and 99.7% respectively) and all were highly related by phylogenetic analyses. Rickettsial DNA in pools of *P. irritans* was not detected.

**Conclusion:**

Our findings highlighted the endemicity of the infection by *R. felis* in fleas from northern of Colombia and showed the likely importance of dogs as hosts of *C. felis felis* fleas and their potential role as reservoirs of *R. felis.*

## Introduction

The genus *Rickettsia* comprised arthropod-associated intracellular and gram-negative bacteria. It is divided into 4 groups based on their genotypic characteristics: Spotted fever group (*R. rickettsii*, *R. conorii*, *R. parkeri*, and several others), typhus group (*R. prowazekii* and *R. typhi*), transitional group (*R. felis*, *R. akari*, and *R. australis*), and the nonpathogenic ancestral group (*R. bellii* and *R. canadensis*) (1). *Rickettsia felis* is globally distributed and is the etiological agent of flea-borne spotted fever. The main vector is the flea *Ctenocephalides felis*, but ticks, mites and lice have also been found infected (2). *Rickettsia felis* in *C. felis* populations is principally maintained by transstadial and transovarial transmission (3). In colonized *C. felis* fleas, vertical transmission of *R. felis* is thought to be the primary route of maintenance, since the reported prevalence of *R. felis* in *C. felis* colonies ranged from 43–100% (4–6). In nature, fleas feeding on *R. felis*-infected mammalian hosts likely amplify the prevalence of *R. felis* in a flea’s population. Studies on the ecology of *R. felis* identified a role for opossums in the transmission cycle (7–9). Furthermore, a role for companion animals, rodents, and, specifically, their fleas as the potential source of human exposure has been suggested (4, 9, 10).

Infecting fleas have been reported in many American countries and human cases of spotted fever by *R. felis* have been recently described in the United States, Mexico, and Brazil (11). Clinical manifestations of flea-borne spotted fever are variable and similar to other Rickettsial diseases. In Colombia, a transversal serological study was performed in seven municipalities of Caldas Province, and a human seroprevalence of 25.2% and 17.8% against *R. typhi* and *R. felis*, respectively was found (12). Additionally, the infection by *R. felis* in *C. felis*, *C. canis*, and *P. irritans* fleas was reported in the province of Caldas (13). Three important spotted fever group (SFG) rickettsiosis outbreaks occurred in Colombia, in the municipalities of Turbo y Necoclí (Antioquia Province) and Los Cordobas (Córdoba Province), between 2006 and 2008 (14, 15). Consequently, these areas have been described as endemic for Rickettsioses in this country.

The purpose of this study was to search for molecular evidence of *Rickettsia* spp*.* in fleas collected from dogs and wild rodents (*Heteromys anomalous*) from the three zones where outbreaks of rickettsioses occurred and discuss the likely role of fleas in the epidemiology of *Rickettsia* spp. in this region of Colombia.

## Materials and Methods

### Study area and sampling

**Fig. 1.**
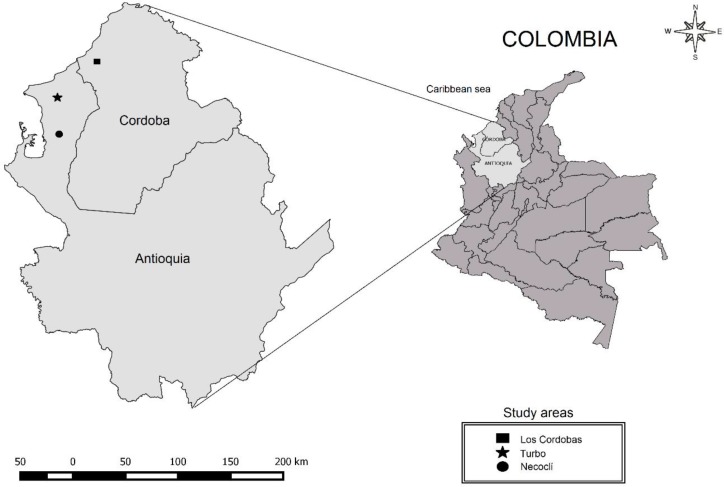
Geographic location of the study areas in northwestern of Colombia. Square corresponds to the municipality of Los Cordobas (Cordoba Province), the star and circle corresponds to the municipalities of Turbo and Necoclí (Antioquia Province), respectively

The study was conducted in 3 neighboring municipalities: Turbo, (8°8.272′N, 76°33.009′ W) located at 400 m above sea level (masl), and Necocli, (8°32.892′N, 76°34.429′W), at 182 m above sea level. Both are located in the Antioquia Province, and Los Cordobas, (8°50.195′N, 76°20.252′W) located at eight meters above sea level, in the Cordoba Province (Fig. 1). All of these municipalities are placed on the Colombian Atlantic Coast. These three sites comprise part of the natural Caribbean region, and have a tropical humid climate characterized by a dry period from Jan to Mar and a rainy season from Apr to Dec, with an annual average temperature of 28 °C and relative humidity of 85% (Fig. 1).

During 2010 and 2012, a total of 649 fleas were removed from 92 dogs of all studied locations (194 from Turbo, 225 from Necocli and 230 from Los Cordobas) and 24 fleas from three *Heteromys anomalus* rodents captured in Turbo and Necocli. They were obtained using tweezers or by combing wild and domestic animals, and care was taken to avoid damaging structures essential for taxonomic classification. The fleas were collected from each animal in one or various vials with alcohol 95% (depending on the number collected per animal) and were transported to the laboratory. Because the population of dogs in the study zones was unknown, a sample size was not determined. However, we were able to estimate the number of animals that lived with people at each site.

Ethical, technical, scientific and administrative standards to perform research in animals were taken into consideration according to national regulations for the procedures of collection, management and conservation of samples (resolution No. 008430 of 1993 and Law 84 of Dec 27^th^ from 1989).

### Molecular detection of *Rickettsia* spp.

Fleas were classified according to morphological keys (16–18). They were grouped in maximum “pools” of 10 individuals, according to host and sampling site: 153 pools of *C. felis felis* were collected from dogs and six pools of *Pulex irritans* fleas were collected from rodents.

DNA from pools was extracted by using QIAamp DNA Mini-Kit (Qiagen®, Valencia, CA, USA), according to manufacturer conditions. Samples were stored at −20 °C until they were used for PCR assays.

Samples were tested by PCR assay with primers CS-78 (forward GCAAGTATCGGTGAGGATGTAAT) and CS323 (reverse GCTTCCTTAAAATTCAATAAATCAGGAT), which amplify a 401bp fragment of the citrate synthase gene (*gltA*), previously reported as appropriate for the screening of *Rickettsia* spp. (19). Samples that came up positive for gltA were tested with the primers Rr190.70p (forward: 5′ATGGCGAATATTTCTCCAAAA)-Rr190.701 (reverse: 3′GTTCCGTTAATGGCAGCATCT), that amplify a 632bp fragment of *ompA* genes (20); primers 120.M59 (forward 5′AAACAATAATCAAGGTACTGT)-120.807 (reverse 3′TACTTCCGGTTACAGCAAAGT) that amplify an 812bp fragment of ompB gene, previously described (21). Negative (molecular grade water) and positive controls (DNA *R. amblyomii*) were included for each reaction. Positive products were purified by using a Quick Gel Extraction kit (PureLink^TM^, Invitrogen) and subsequently these were sequenced by a commercial facility (Macrogen). The sequences were assembled and edited with the Seqman program from the DNAstar packet (Lasergene®, Madison WI, USA), and phylogenetic analysis was performed with the MEGA 6 (22) and MrBayes 3.2 programs (23).

## Results

**Fig. 2.**
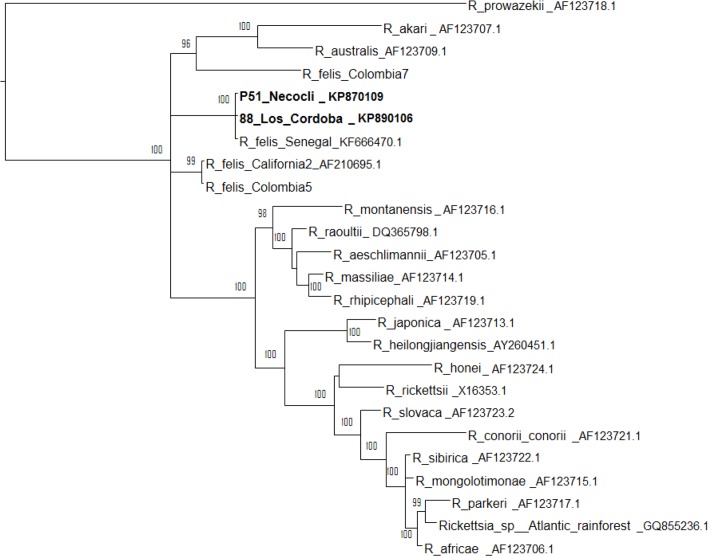
Bayesian phylogenetic tree of rickettsial ompB gene. Samples from this study are shown on bold font. Two parallel searches were runned by 1,000,000 generations sampling every 1,000 states. Average standard deviations of split frequencies were <0.01 at the end of the runs. Substitution model used was the General Time Reversible with a discrete Gamma distribution of the variation of the evolutionary rate (GTR+G), previously found as the best model by the Bayesian information criterion. The analysis was performed in MrBayes 3.2.0. There were a total of 723 positions in the final dataset. The tree was drawn in the software, 1.4 and it was rooted with *R. prowazekii.*

Of 153 pools of *C. felis felis* (54 were from Turbo, 65 from Necocli and 34 from Los Cordobas), Rickettsial DNA was detected in 30 (19%) pools by gltA gene. Four pools amplified for ompB gene and none amplified for ompA. *Pulex irritans* pools were negative by PCR. The prevalence of *Rickettsia* in fleas expressed as percentage and minimum infection rate (MIR) of fleas were calculated. We made this assessment on the assumption that a PCR-positive pool contains only one positive specimen. The overall MIR of infected fleas was 4.45 (30/673). Of these, 4.6% (9/194) of *C. felis* was from Turbo, 5.7% (13/225) from Necocli and 3.5% (8/230) from Los Cordobas (Table 1). Nucleotide sequences of the ompB gene from Necocli and Los Cordobas were 99.9% identical to each other (Fig. 2). Sequence homology obtained from Necocli and Los Cordobas were 100 and 99.7% with *R. felis* strain Senegal, respectively. Evolutionary history of gltA gene was inferred by using the Neighbor-Joining method (not shown) and the Bayesian method was used for ompB gene (Fig. 2).

**Table 1.** Prevalence of *Rickettsia felis* in fleas expressed as the minimum percentage of fleas in a pool with detectable *Rickettsiae* and Minimum Infection Rate (MIR) (IC 95%)

**Location**	**Species of fleas**	**Number Fleas**	**Number Pools positive / Number tested pools**	**% positives pools**	**Minimum Infection Rate (MIR)**
**Turbo**	*C. felis felis*	194	9/54	16	9/194 (4.6%)
	*Pulex irritans*	5	0/2	0	0/5 (0%)
**Necoclí**	*C. felis felis*	225	13/65	20	13/225 (5.7%)
	*Pulex irritans*	19	0/4	0	0/19 (0%)
**Los Córdobas**	*C. felis felis*	230	8/34	23	8/230 (3.5%)

	Total fleas pools	673	30/159	19	30/673 (4.4%)

The sequences generated in this study have been submitted to GenBank under the accessions KP870106 to KP870109.

## Discussion

We reported the infection by *R. felis* in *C. felis felis* fleas collected from dogs from endemic areas of rickettsioses in Cordoba and Antioquia provinces, northern of Colombia. Values of minimum infection rates (MIR) reported herein for *C. felis* are similar to the one previously reported in the province of Caldas, Colombia (5.3% MIR) (13). However, there are lower than MIRs shown in other countries, such as Brazil (14.3%) (24), the United States (13.3%) (25) and Taiwan (8.2%) (26).

The proportion of *C. felis felis* positive pools in our study was 19% (30/153) and the proportion obtained in Caldas (Colombia) was 41% (54/132) (13). The rates of MIRs of these studies were calculated based on the assumption that only one flea from each positive pool was positive for the *Rickettsia* gene evaluated. It may underestimate the frequency of *R. felis* in pools, possibly because of the greater amount of DNA in pools or other contaminants that may inhibit PCR assays (27). Otherwise, in Brazil, differences in the percentage of infection between regions were related to the environmental and climatic conditions (28). Higher rates of *R. felis* infection in fleas were significantly related with regions with temperate climates, and lower rates were linked with dry climates.

Several studies highlight the broad distribution of infection by *R. felis* in *C. felis*. Different proportions of infection have been reported in other American countries. For example, in Mexico, 20% of 54 pools of *C. felis* collected from dogs were reported infected (29). Sixty-four percent (55/86) and 58% (47/81) of pools of *C. felis* removed from cats and dogs were infected in Guatemala and Costa Rica, respectively (30); and 41% of infected pools (25/62 *C. felis* and 2/4 *C. canis*) collected from 15 cats and dogs were reported in Uruguay (31). In our study, *R. felis* was detected in 30/153 (19%) *C. felis* pools removed from dogs, which is very similar to the Mexican report which, by the way, suggests the likely relevance of this host in maintaining *C. felis* and possibly *R. felis* in studied areas. Moreover, some studies have detected *R. felis* by PCR in blood of dogs, suggesting that dogs may have the potential to act as an important reservoir of infection (32, 33).

In the present study, the sequences obtained from Necocli and Los Cordobas were identical to each other and they showed extremely high sequence homology to a *R. felis* strain from Senegal (100 and 99.7% respectively, Fig. 2). In province of Caldas (Colombia), authors have described a high homology (>98%), between several *R. felis* sequences obtained from *C. felis* and the *R. felis* URRWXCal2 (Genbank accession CP 000053). Likewise, they showed a very close monophyletic relationship of these sequences with the *R. akari* group (13).

Sequences of *R. felis* from Necoclí (KP 870109) and Los Cordobas (KP870106) obtained in the present study were compared with the sequences obtained in Caldas (Colombia), called Colombia5 and Colombia7 (Fig. 2) (13). Phylogenetic relationship between the sequences of our results and Caldas (Colombia), showed identity values of 95.9% of Necoclí vs Colombia5; and 92.7% of Los Cordobas vs Colombia7. Variations could exist between the sequences of strains of *R. felis* from two different areas of Colombia. Moreover, Rickettsial DNA was not detected in *P. irritans* pools of our study, in contrast, 3/10 pools of this species infected with *R. felis* in Caldas were reported (Colombia) (13). *R. felis* infection in *P. irritans*, has also been reported in studies from Democratic Republic of the Congo (34) and the United States (4), that shows a likely wide distribution of *R. felis* in this fleas species around the world.

Human infection with *R. felis* and its clinical implications have been controversial. This microorganism may be an emerging human pathogen; meanwhile, other authors consider that their casual appearance in human samples and vector is a proof of endosymbiosis (11, 35, 36). Before we determine whether human beings of this region of Colombia could be at real risk of getting ill by *R. felis*, further studies are necessary to show the seroprevalence in humans and animals and demonstrate its presence in other human cases compatible with rickettsiosis.

## Conclusion

In the present study, we reported the infection by *Rickettsia felis* in *C. felis felis* fleas collected from dogs from endemic areas of rickettsioses in Cordoba and Antioquia provinces (Colombia). Almost 20% (30/153) of *C. felis felis* pools contained Rickettsial DNA. Our findings highlighted the endemicity of the infection by *R. felis* in fleas from northern of Colombia and suggest the importance of dogs as host of *C. felis felis* fleas and their potential as reservoirs of *R. felis*. Human infection with *R. felis* and its clinical implications have been controversial. May before we determine whether human beings of this region of Colombia could be at real risk of getting ill by *R. felis*, further studies are necessary to show the seroprevalence in humans and animals and demonstrate its presence in other human cases compatible with rickettsiosis.
